# *Paraburkholderia phymatum* Homocitrate Synthase NifV Plays a Key Role for Nitrogenase Activity during Symbiosis with Papilionoids and in Free-Living Growth Conditions

**DOI:** 10.3390/cells10040952

**Published:** 2021-04-20

**Authors:** Paula Bellés-Sancho, Martina Lardi, Yilei Liu, Sebastian Hug, Marta Adriana Pinto-Carbó, Nicola Zamboni, Gabriella Pessi

**Affiliations:** 1 Department of Plant and Microbial Biology, University of Zürich, CH-8057 Zürich, Switzerland; paula.belles@botinst.uzh.ch (P.B.-S.); lardimartina@gmail.com (M.L.); yilei.liu@botinst.uzh.ch (Y.L.); sebastian.hug@uzh.ch (S.H.); marta.pinto@botinst.uzh.ch (M.A.P.-C.); 2 ETH Zürich, Institute of Molecular Systems Biology, CH-8093 Zürich, Switzerland; zamboni@imsb.biol.ethz.ch

**Keywords:** rhizobia, papilionoid, mimosoid, nodule, gene expression, metabolomics

## Abstract

Homocitrate is an essential component of the iron-molybdenum cofactor of nitrogenase, the bacterial enzyme that catalyzes the reduction of dinitrogen (N_2_) to ammonia. In nitrogen-fixing and nodulating alpha-rhizobia, homocitrate is usually provided to bacteroids in root nodules by their plant host. In contrast, non-nodulating free-living diazotrophs encode the homocitrate synthase (NifV) and reduce N_2_ in nitrogen-limiting free-living conditions. *Paraburkholderia phymatum* STM815 is a beta-rhizobial strain, which can enter symbiosis with a broad range of legumes, including papilionoids and mimosoids. In contrast to most alpha-rhizobia, which lack *nifV*, *P. phymatum* harbors a copy of *nifV* on its symbiotic plasmid. We show here that *P. phymatum nifV* is essential for nitrogenase activity both in root nodules of papilionoid plants and in free-living growth conditions. Notably, *nifV* was dispensable in nodules of *Mimosa pudica* despite the fact that the gene was highly expressed during symbiosis with all tested papilionoid and mimosoid plants. A metabolome analysis of papilionoid and mimosoid root nodules infected with the *P. phymatum* wild-type strain revealed that among the approximately 400 measured metabolites, homocitrate and other metabolites involved in lysine biosynthesis and degradation have accumulated in all plant nodules compared to uninfected roots, suggesting an important role of these metabolites during symbiosis.

## 1. Introduction

Nitrogen in the form of dinitrogen gas (N_2_) comprises approximately 78% of the Earth’s atmosphere. However, dinitrogen is metabolically inaccessible for plants, which makes it a limiting factor for crop production [1,2]. During the past century, the agricultural industry has mainly relied on synthetic fertilizers in order to obtain higher crop yields. Their substantial contribution to environmental pollution has led to an urgent search for more environmentally sustainable alternatives [3,4,5]. One promising and agriculturally important alternative to fertilizer usage is biological nitrogen fixation (BNF), whereby rhizobia reduce N_2_ inside specialized root or stem organs (nodules) and feed the legume with combined nitrogen [6]. Rhizobia are polyphyletic and are represented by species in 18 genera of the alpha-proteobacteria (so called alpha-rhizobia) and beta-proteobacteria (beta-rhizobia) [7,8,9,10,11,12,13]. Phylogenetic analyses based on the conservation of genes involved in N_2_ reduction and nodulation (*nif* and *nod* genes, respectively) proposed an independent evolution of these subclasses of rhizobia, which would suggest that different molecular mechanisms are used to establish symbiosis [10,13,14,15]. Currently, the beta-rhizobial group includes several *Burkholderia* species that have been re-classified into the new genera *Paraburkholderia* [8,16] and *Trinikia* [17]. The *Paraburkholderia* genus is composed of mostly plant-beneficial bacteria and includes strains which are able to perform BNF. The diazotrophic *Paraburkholderia* strains encompass symbiotic legume nodulating strains and non-nodulating strains that fix N_2_ under free-living growth conditions (free-living diazotrophs). Phylogenetical analysis based on the nodulation (*nod*) genes has divided the nodulating group into *Paraburkholderia* nodulating mimosoids and *Paraburkholderia* nodulating papilionoids [18]. *Paraburkholderia phymatum* STM815 [10] is an exception to this rule. This strain is classified as a *Paraburkholderia* nodulating mimosoids [19] and has indeed been isolated from several different *Mimosa* spp. [20,21,22,23,24,25]. However, *P. phymatum* is also able to establish nitrogen-fixing symbioses with papilionoids and to reduce nitrogen in free-living conditions [22,26,27].

Inside root nodules, rhizobia rely on carbon sources from the host plant for growth and energy, allowing them to convert atmospheric nitrogen (N_2_) into ammonium (NH_3_) through the enzyme nitrogenase. The root nodules provide a microoxic environment, which is essential for the activity of the nitrogenase [28]. The nitrogenase consists of two proteins, the dimeric reductase (also known as Fe protein, encoded by *nifH*) and the heterotetrameric dinitrogenase (or MoFe protein, encoded by *nifD* and *nifK*). The MoFe protein contains the catalytic site of the enzyme and two types of metal centers: the P-cluster and the MoFe cofactor (FeMo-co), which carries homocitrate as a ligand [28,29,30]. Free-living diazotrophs can synthetize homocitrate through NifV, a homocitrate synthase that catalyzes the condensation of acetyl coenzyme A and 2-oxoglutarate into homocitrate. The free-living diazotrophs *Klebsiella pneumoniae* (reclassified as *K. sp.* M5Ia [31]) and *Azotobacter vinelandii* have been shown to depend on the presence of *nifV* to reduce N_2_ to ammonia [32]. Conversely, most nodulating symbiotic alpha-rhizobia lack *nifV* in their genome and only display an efficient nitrogen fixation when they are in symbiosis with the host plant. During the *Mesorhizobium loti–Lotus japonicus* symbiosis, the absence of *nifV* in *M. loti* is compensated by the provision of homocitrate from the plant through the nodule-specific homocitrate synthase FEN1 [33]. However, in the nitrogen-fixing symbiosis between the photosynthetic *Bradyrhizobium* ORS285 strain and the Nod factor-independent *Aeschynomene* plants, the lack of *nifV* was not complemented by the plant, demonstrating that not all legumes supply the bacterial partner with sufficient homocitrate to allow for efficient nitrogen fixation [34]. The beta-rhizobium *P. phymatum* carries a single *nifV* gene on its symbiotic plasmid (Bphy_7741), and previous studies from our laboratory revealed that *nifV* was among the top 200 upregulated genes during symbiosis with *Phaseolus vulgaris* compared to aerobic free-living growth conditions [35].

The objective of this work was to characterize the role of *P. phymatum* STM815 NifV both in symbiosis with different papilionoid and mimosoid legumes and during free-living growth. Our results suggest that *nifV* is essential for nitrogenase activity during symbiosis with papilionoid legumes and in oxygen-limited free-living conditions. Although the bacterial *nifV* gene was highly expressed in all host plants, it was dispensable during symbiosis with *Mimosa pudica*. Finally, to understand the metabolic differences between mimosoid and papilionoid nodules, we performed metabolomics from nodules and uninfected roots of the tested papillionoid and mimosoid plants. Analysis of the occurring metabolic changes of close to 400 metabolites revealed an accumulation of homocitrate and intermediates from lysine, arginine and proline metabolism in all nodules compared to the respective non-infected roots.

## 2. Materials and Methods

### 2.1. Bacterial Strains, Media and Cultivation

The bacterial strains, plasmids and primers used in this work are listed in Supplementary Table S1. *E. coli* strains were routinely cultured aerobically in Luria-Bertani liquid medium (LB) [36], whereas *P. phymatum* STM815 strains were grown in a LB liquid medium without salt [35] and in ABS (AB minimal media [37] with 15 mM succinic acid as a carbon source) [38]. Where necessary, the following antibiotics were used: chloramphenicol (20 µg/mL for *E. coli* and 80 µg/mL for *P. phymatum*), kanamycin (25 µg/mL for *E. coli* and 50 µg/mL for *P. phymatum*) and tetracycline (15 µg/mL for *E. coli* and 30 µg/mL for *P. phymatum*). For the in vitro nitrogenase activity test, a variation of the (A)BS minimal media without nitrogen source was utilized [38], with the addition of 0.4% agarose. In order to create nitrogen-limited conditions, (A)BS medium was supplemented with 0.3 mM NH_4_Cl and nitrogen-replete growth was achieved using 15 mM NH_4_Cl.

### 2.2. Phylogenetic Analysis

To find NifV homologous proteins in the *Paraburkholderia* genus (Supplementary Table S2), the amino acid sequence of NifV (Bphy_7741) from *P. phymatum* STM815 was blasted (BLASTP) against the Integrated Microbial Genomes and Microbiomes (IMG) database from the Joint Genome Institute (JGI) [39]. In this analysis, only the genome of one representative strain of each *Paraburkholderia* species available in the JGI IMG database was selected. Filtering criteria applied for homolog identification were at least 70% of identity at the protein level and an e-value lower than 1 × 10^−5^ (Supplementary Table S3). The NifV protein sequences collected from the JGI IMG database were aligned with ClustalW [40] and the phylogenetic analysis was conducted using the Jones-Taylor-Thornton model with five categories of gamma rate distribution. *Klebsiella sp.* M5aI NifV was used as an outgroup. For the strains containing *nifV*, their 16S rRNA nucleotide sequences were obtained from the National Center for Biotechnology Information (NCBI) GenBank database. Those rRNA sequences were aligned with MUSCLE [41] and the phylogenetic reconstruction was done using the Tamura-Nei parameter model following a discrete gamma distribution with five rate categories and invariable sites. For this analysis, *Bradyrhizobium* sp. ORS285 was used as the outgroup. The alignment trimming and maximum likelihood phylogenetic reconstruction were performed with MEGA X with 100 bootstrap replicates [42].

### 2.3. Construction of P. phymatum STM815 Mutant Strains

The genomic DNA (gDNA) of *P. phymatum* STM815 was isolated using the GenElute^TM^ Bacterial Genomic DNA Kit (Sigma-Aldrich, St. Louis, MO, United States), while plasmid DNA from *E. coli* strains was obtained by using the QIAprep Spin Miniprep Kit (Qiagen, Hilden, Germany). To generate the deletion mutant strains, two external fragments flanking the *nifV* gene were used as recombination sites for the deletion. The 485 bp-long upstream fragment was amplified using the primers Bphy7741_up_F_EcoRI and Bphy7741_up_R_NdeI, and for the amplification of the 479 bp-long downstream fragment, the Bphy7741_down_F_NdeI and the Bphy7741_down_R_EcoRI primers were used (Supplementary Table S1). For amplification, the Phusion High-Fidelity DNA polymerase (ThermoFischer, Waltham, MA, USA) was employed. Both fragments were digested with *Nde*I and ligated. The ligated DNA product was amplified using the Bphy7741_up_F_EcoRI and Bphy7741_down_R_EcoRI primers, then digested with *EcoR*I and cloned into the pEX18Tc vector. The chloramphenicol resistance cassette (Cm^R^) was amplified with the primers catA2_F_NdeI and catA2_R_NdeI using the pSHAFT2 vector as a template, digested with *Nde*I and inserted between the two upstream and downstream fragments in the pEX18Tc vector in either forward or reverse direction. The constructed plasmids were transferred into wild-type *P. phymatum* by triparental mating. The transconjugants were purified and replica-plated on selective minimal medium. The genomic integration as well as the orientation of the Cm^R^ was verified by PCR using the Bphy7741_veri and the Bphy7742_veri primers in combination with the catA2_F_NdeI and catA2_R_NdeI primers, respectively. Importantly, the two mutants differ on the direction of the inserted Cm^R^: while in the so-called *nifV* forward mutant, the Cm^R^ with its promoter is located in the same orientation as the downstream genes, in the *nifV* reverse mutant, the Cm^R^ is placed in the opposite orientation (Supplementary Figure S1). To complement the *nifV* mutant phenotype, *P. phymatum nifV* was introduced in *trans* into the *nifV* forward mutant strain. The gene was amplified by PCR using the primers Bphy7741_comp_F_XbaI and Bphy7741_comp_R_HindIII. The 1457 bp-long product was digested with *Xba*I and *Hind*III and cloned into the pBBR1MC2 vector. The complementing plasmid was mobilized into the corresponding mutant strain by triparental mating and the resulting strain was called *P. phymatum nifV* forward complemented strain. The construction of the green fluorescence protein (GFP) expressing reporter strain was carried out by introducing the *nifV* promoter sequence into the vector pPROBE-NT. The promotor of the *nifV* gene was amplified from *P. phymatum* STM815 gDNA with the primers Bphy7741_prom_EcoRI_F and Bphy7741_prom_SalI_R. The PCR product, with a length of 431 bp, was digested with *EcoR*I and *Sal*I and cloned into pPROBE-NT. The plasmid was transferred by triparental mating into *P. phymatum* STM815 wild-type strain. The correct sequences of all the constructed plasmids were confirmed by sequencing at Microsynth (Balgach, St. Gallen, Switzerland).

### 2.4. Nitrogen-Fixation In Vitro Test

The ability of *P. phymatum* STM815 and mutant strains to reduce nitrogen in free-living conditions was tested by using the acetylene reduction assay (ARA). Pre-cultures of the tested strains were grown in LB media without salt, washed twice, and inoculated into glass tubes containing 7 mL soft-agarose medium (0.4% agarose) to reach a final OD_600_ of 0.05 just before the medium solidified. The medium used in the tests was (A)B minimal media with 15 mM of succinic acid ((A)BS) as a carbon source and either 15 or 0.3 mM of NH_4_Cl as a nitrogen source. For chemical complementation assays, 1 mM of sodium (±)-homocitrate tribasic (Sigma-Aldrich) was added to the media. After inoculation, the glass tubes were closed with silicon septum stoppers and 1 mL of acetylene (PanGas, Zurich, Switzerland) was injected. The tubes were incubated at 28 °C for 8 weeks and acetylene reduction was measured by gas-chromatography (Agilent Gas Chromatograph 7820A, Agilent Technologies) every 7 days for 2 months. At the end of the incubation, the cells were re-isolated from the tubes by adding one volume of 10 mM MgSO_4_. At least three independent biological replicates per condition and strain were used in this assay. The number of cells was determined by colony forming units (CFU) on LB medium without salt plates and the identity of the strains was verified by PCR using the respective primers (Supplementary Table S1).

### 2.5. Plant Growth Condition, Inoculation and Plant Harvesting

Seeds of the common bean (*Phaseolus vulgaris*, cv. Negro jamapa) and cowpea (*Vigna unguiculata* (L.) Walp. cv. Red Caloona) were surface-sterilized with absolute ethanol and a 35% solution of H_2_O_2_, as previously described [26]. Siratro (*Macroptilium atropurpureum*) and mimosa (*Mimosa pudica*, Samen Mauser AG commercial seeds) seeds were surface-sterilized with a 96% H_2_SO_4_ solution and a 3% NaClO solution, as previously described [25]. After germination, the seeds were transferred into autoclaved amber yogurt jars containing vermiculite (VTT-Group, Muttenz, Switzerland) and Jensen medium without nitrogen [43]. The plants were inoculated with a final OD_600_ of 0.025 for each strain, as we previously reported [38]. The plants were grown with a temperature of 22 °C at night and 25 °C during the day, and light (200 µM light intensity) was supplied for 16 h and the humidity was constantly set to 60%. The root nodules were harvested for further analysis when the nitrogenase activity was maximal: 21 days post-inoculation (dpi) for common bean and cowpea plants, and 28 dpi for siratro and mimosa. To analyze the root nodule bacteroids, firstly, roots were surface-sterilized as previously described [44]. The bacteroids in the nodule were released by crushing the nodule in 100 µl LB liquid medium without salt with 25% glycerol, and the number of cells (nodule occupancy) was determined on LB medium without salt plates supplemented with the correspondent antibiotic. The identity of the strains was verified by PCR using the respective primers (Supplementary Table S1). The nitrogenase activity was measured by the ARA assay as previously described [35,45]. Next, the nodules were dried overnight at 60 °C. At least two independent biological replicates with at least four plants per strain were performed per plant type. To observe the expression of the *nifV* promoter in nodules, the *nifV*-pPROBE-NT reporter strain as well as *P. phymatum* containing an empty pPROBE-NT were tested in planta. Three independent biological replicates with one plant per strain were used for microscopic analysis. For metabolomics experiments, common bean, cowpea, siratro and mimosa were inoculated with *P. phymatum* STM815 wild-type strain, and for metabolites comparison, nodules and uninfected roots of these four host plants were collected. Nodules and uninfected roots were harvested 21 or 28 dpi. Three biological replicates were analyzed per infected and uninfected host plant, except for the uninfected roots from common bean, where only duplicates were used.

### 2.6. Microscopy and Image Analysis

Nodules were collected from the roots and washed with deionized water. Next, the nodules were cut into 1 mm thick sections using a clean blade and placed on a glass slide. Fluorescence microscopy of nodule sections and acquisition of the pictures were done using a confocal laser scanning microscope (DM5500Q; Leica) equipped with a ×40/1.3 objective as well as a detector and filter sets for the monitoring of GFP (488 nm for excitation and emission at 501–540 nm). For the visualization of *nifV* expression when *P. phymatum* wild-type was grown in vitro, images of the tubes were taken every 7 days for 21 days using a custom-built fluorescence imaging device (Infinity 3 camera, Lumenera, Canada) with an excitation/emission wavelength of GFP at 490 nm/510 nm. Captured images were analyzed using ImageJ2 (Fiji distributions) [46].

### 2.7. Metabolite Extraction and Metabolomics Data Analysis

For metabolites extraction, between 20 and 30 mg (fresh weight) of nodules or roots were processed per sample. After harvesting, the nodules and the roots were immediately flash-frozen in liquid nitrogen. Frozen nodules and roots were crushed with one 3 mm tungsten bead (TissueLyser II, QIAGEN). Afterwards, two mL of 80% cold methanol were added for metabolites’ extraction. The samples were stored at −20 °C for 20 min with regular mixing. Finally, the samples were flash-frozen in liquid nitrogen [47]. The extracts were injected twice and analyzed by non-targeted flow injection-time-of-flight mass spectrometry on an Agilent 6546 QTOF instrument (Agilent Technologies, Santa Clara, CA, USA). The used settings are equivalent to the ones described previously [47]. Data processing was done with custom software coded in Matlab (Mathworks) and Python [48]. Detected ions were putatively annotated on the basis of accurate mass and isotopic patterns [48] and against the aggregated list of metabolites associated to *P. phymatum*, *P. vulgaris*, or *V. unguiculata* according to the KEGG database. Within a *m*/*z* tolerance of 0.001 Da, a total of 384 ions were matched to expected deprotonated, monoisotopic molecules. The complete list is reported in Supplementary Table S4.

### 2.8. Statistical Analysis

The statistical analysis of nitrogenase activity measurements and of the plant symbiotic properties was performed with GraphPad Prism 6.0 using an ANOVA and Tukey’s test. Significant differences between the amounts of cells (CFU) isolated from the in vitro nitrogen fixation test were analyzed with Microsoft Excel (2016) using a paired *t*-test. For the comparative statistical analysis of the metabolomics data, an unpaired *t*-test was applied and the obtained *p*-values were adjusted using the Benjamini-Hochberg procedure [49]. The *q*-values were calculated according to Storey and Tibshirani [50].

## 3. Results

### 3.1. NifV Is Widely Distributed in Paraburkholderia Strains

**Figure 1:**
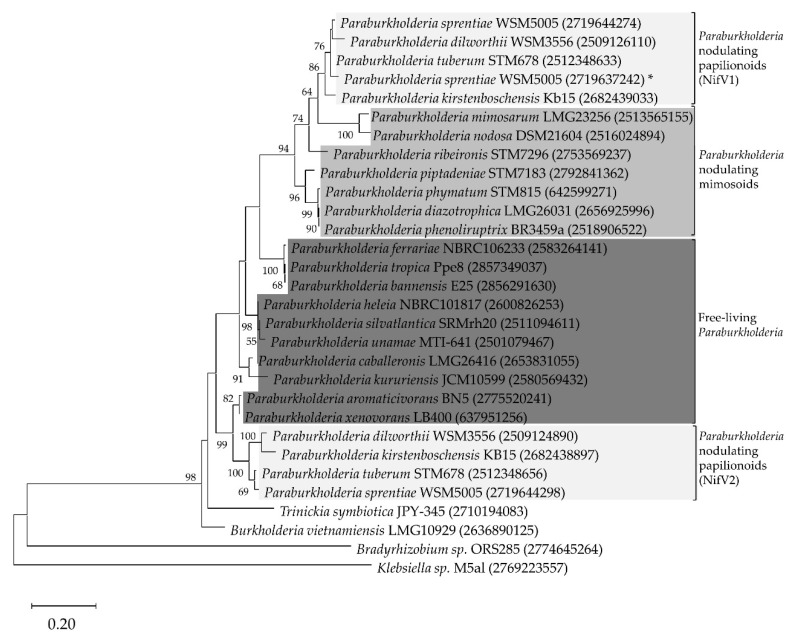
Maximum likelihood phylogenetic tree of nitrogen-fixing beta-proteobacteria strains based on NifV amino acid sequences. Colored boxes indicate the different *Paraburkholderia* groups and the corresponding NifV copy is shown after the name group (NifV1 or NifV2). The asterisk indicates the third NifV copy found in *P. sprentiae* (*). Bootstrap test values after 100 replicates are expressed as percentages and values are shown if greater than 50%. The respective IMG gene identification number is shown in brackets after the species name.

A BLASTP search using *P. phymatum* STM815 NifV revealed homologs in 21 out of the 48 representative *Paraburkholderia* species whose draft genome were available in the JGI IMG database (Supplementary Table S2). In line with de Meyer et al. [18], who had grouped *Paraburkolderia* according to their nodulation characteristics, we classified those 21 strains in three different groups (Figure 1): free-living *Paraburkholderia* strains, which do not nodulate legumes (*P. aromaticivorans* BN5, *P. bannensis* E25, *P. caballeronis* LMG26416, *P. ferrariae* NBRC106233, *P. heleia* NBRC101817, *P. kururiensis* JCM10599, *P. silvatlantica* SRMrh20, *P. tropica* Ppe8, *P. unamae* MTI-641, *P. xenovorans* LB400), *Paraburkholderia* nodulating mimosoid plants (*P. diazotrophica* LMG26031, *P. mimosarum* LMG23256, *P. nodosa* DSM21604, *P. phenoliruptrix* BR3459a, *P. phymatum* STM815, *P. piptadeniae* STM7183, *P. ribeironis* STM7296) and *Paraburkholderia* nodulating papilionoid plants (*P. dilworthii* WSM3556, *P. kirstenboschensis* Kb15, *P. sprentiae* WSM5005, *P. tuberum* STM678). In all *Paraburkholderia* nodulating papilionoids, we found either two (for *P. dilworthii* WSM3556, *P. kirstenboschensis* Kb15 and *P. tuberum* STM678) or three (for *P. sprentiae* WSM5005) copies of *nifV*. For all these strains, we observed amino acid identities above 70% among the two or three NifV copies.

**Figure 2:**
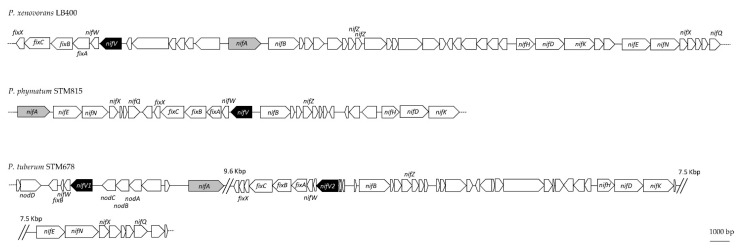
Genomic location of *nifV* in representative *Paraburkholderia* strains. *P. xenovorans* LB400, *P. phymatum* STM815 and *P. tuberum* STM678 as representatives of the groups free-living *Paraburkholderia*, *Paraburkholderia* nodulating mimosoids and *Paraburkholderia* nodulating papilionoids, respectively. The *nifV* gene is highlighted in black and the transcriptional regulator *nifA* in grey.

These results were compared with a 16S rRNA-based phylogenetic tree, where *Bradyrhizobium* sp. ORS285 was used as the outgroup. In both reconstructions, the *Paraburkholderia* strains were grouped together (Supplementary Figure S2). In the NifV maximum likelihood tree, the *Paraburkholderia* nodulating papilionoids and mimosoids were grouped separately from the free-living *Paraburkholderia*. The phylogenetic reconstruction showed the NifV copy of the *Paraburkholderia* strains nodulating papilionoids in a different monophyletic group, one close to the *Paraburkholderia* nodulating mimosoids (bootstrap value of 64%) and the other group nested with the free-living (bootstrap value of 99%) (Figure 1). Interestingly, for the *Paraburkholderia* strains nodulating papilionoids, the position of all *nifV* copies in the *nif* cluster was conserved: the *nifV* copy exhibiting similarity to *nifV* of nodulating mimosoids (*nifV*1) was located upstream of the regulatory *nifA* gene (key transcriptional regulator for nitrogenase activity), while the second *nifV* copy more similar to *nifV* of the *Paraburkholderia* free-living group (*nifV*2) was located downstream of *nifA* (Figure 2). However, the third *nifV* copy in *P. sprentiae* WSM5005 was localized outside of the *nif* cluster. In all the three strains used for the reconstruction of the *nifV* genomic location (*P. xenovorans* LB400, *P. phymatum* STM815 and *P. tuberum* STM678), *nifV* is predicted to be located at the beginning of an operon together with the nitrogenase-stabilizing *nifW* gene and the *fixABCX* genes involved in electron transport (Figure 2, Supplementary Figure S4). These results suggested that the distribution of the different *nifV* copies as well as their genomic localization are conserved among members of the three diazotrophic *Paraburkholderia* groups (free-living, nodulating papilionoids and nodulating mimosoids).

### 3.2. P. phymatum nifV Is Essential for Nitrogenase Activity in Symbiosis with Papilionoid Host Plants

**Figure 3:**
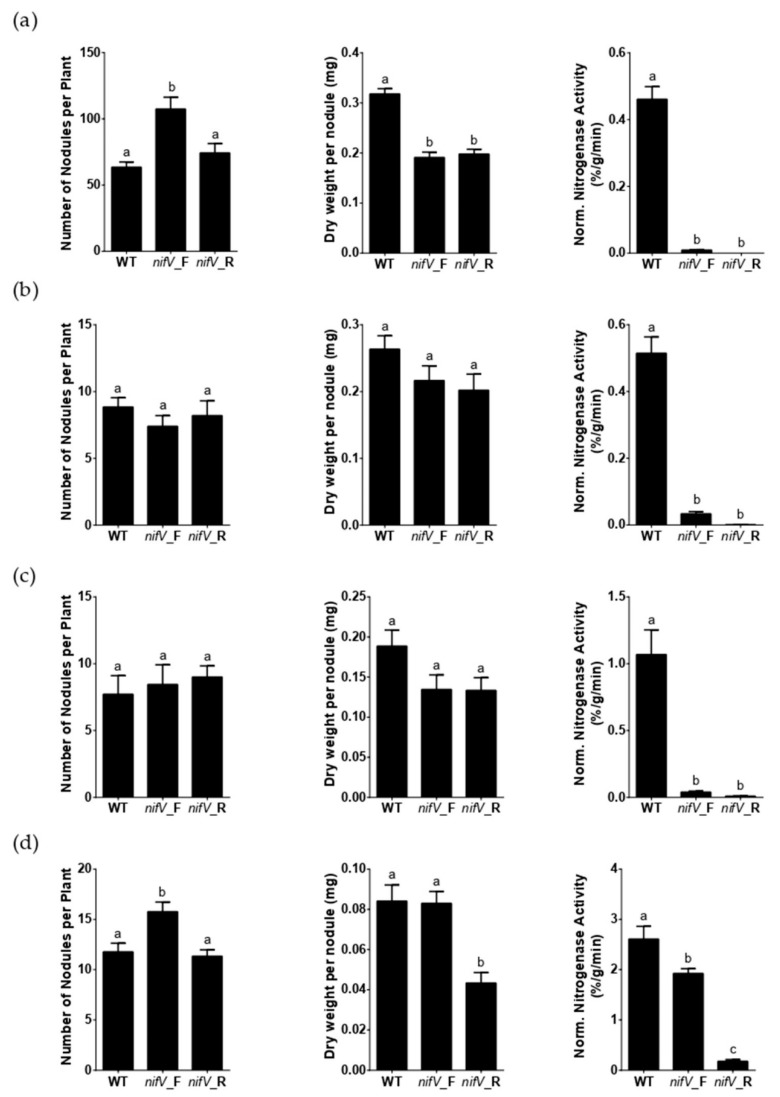
Symbiotic properties of (**a**) *Phaseolus vulgaris* (common bean), (**b**) *Vigna unguiculata* (cowpea), (**c**) *Macroptilium atropurpureum* (siratro) and (**d**) *Mimosa pudica* in symbiosis with different *P. phymatum* strains: wild-type (wt), *nifV* forward (*nifV*_F) and reverse (*nifV*_R) mutants. Number of nodules per plant, dry weight per nodule and normalized nitrogenase activity on day 21 or 28 after inoculation is given. Error bars indicate the standard error of the mean (SEM). For each histogram, values with the same letter (a, b, c) are not significantly different, while those with different letters are (ANOVA, Tukey’s test with *p*-value ≤ 0.05).

The importance of *P. phymatum* STM815 *nifV* during symbiosis was tested using four leguminous plants that can establish a nitrogen-fixing symbiosis with *P. phymatum*. Three legumes belong to the subfamily of papilionoids (*Phaseolus vulgaris* cv. Negro jamapa, *Vigna unguiculata* (L.) Walp. cv. Red Caloona and *Macroptilium atropurpureum*) and one to the subfamily of mimosoids (*Mimosa pudica*). The different seeds were inoculated with 10^7^ CFU of *P. phymatum* wild-type or mutant strains. Importantly, since *nifV* is the first gene in an operon with *nifW* and *fixABC*X (Supplementary Figure S1), we created two *nifV* deletions mutant strains by introducing the antibiotic resistance cassette in both reverse and forward direction with respect to the direction of transcription of the *nifV* gene (see Material and Methods Section, and Supplementary Figure S1). After three weeks of incubation, the nodules induced by both *nifV* mutant strains in the three papilionoid plants exhibited a very low nitrogenase activity: between 2.2% and 5.9% of the wild-type nitrogenase activity for the *nifV* forward mutant and between 0% and 0.93% for the *nifV* reverse mutant. Furthermore, 6.9% of the wild-type nitrogenase activity was observed in mimosa nodules containing the *nifV* reverse mutant (Figure 3a–d). In contrast, mimosa nodules occupied by the *nifV* forward mutant strain (which contains the antibiotic resistance cassette in the same orientation as the genes in the operon) showed 73.6% of the nitrogenase activity measured in nodules infected with the wild type. The roots of common bean and mimosa plants inoculated with the *nifV* forward mutant showed a significantly increased number of nodules (Figure 3a,d). Moreover, common bean nodules occupied by the wild-type strain showed a significantly higher dry weight per nodule compared to the nodules inoculated with both *nifV* mutant strains (*nifV* forward and reverse). The nodule occupancy and the identity of the bacteroids was confirmed from one nodule of each plant species for each biological replicate. As shown in Supplementary Figure S3, wild-type and *nifV* mutant strains occupied the nodules of all four plants in similar numbers. The results obtained with the *nifV* forward mutant strain indicated that *nifV* is essential for nitrogenase activity in papilionoids but not in the mimosoid legume tested.

### 3.3. P. phymatum nifV Is Essential for Nitrogenase Activity in Free-Living Conditions

**Figure 4:**
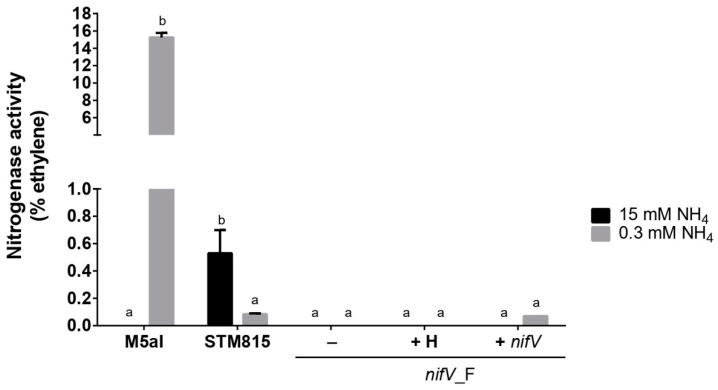
Nitrogenase activity in *Klebsiella sp.* M5aI (M5aI), *P. phymatum* wild-type (STM815), *nifV* mutant strain (*nifV*_F) and complemented strains, either genetically complemented (+*nifV*) or chemically complemented with 1 mM of homocitrate (+H) 21 days post-inoculation. The strains were grown in (A)BS soft agarose tubes with different concentrations of the nitrogen source: 15 mM NH_4_Cl and 0.3 mM NH_4_Cl (3 biological replicates each). Error bars indicate the standard error of the mean (SEM). For each strain, values with the same letter (a, b) are not significantly different, while those with different letters are (ANOVA, Tukey’s test with *p*-value < 0.05).

The nitrogenase activity of *P. phymatum* wild type, *nifV* mutant and complemented strains during free-living growth conditions was determined in tubes containing (A)B minimal medium with 15 or 0.3 mM of ammonia and succinic acid as a carbon source. The tubes were inoculated with an initial OD_600_ of 0.05 (approximately 10^7^ CFU) and then tightly closed in order to create a microoxic environment, which is essential for nitrogenase activity. The free-living diazotroph *Klebsiella sp.* M5aI was used as a positive control since this strain can efficiently reduce atmospheric nitrogen, specifically when grown under nitrogen limitation. High concentrations of nitrogen are known to inhibit the expression of the *Klebsiella sp.* M5aI genes involved in nitrogenase activity [51]. After 21 days of incubation at 28 °C, *P. phymatum* wild type was the only strain showing nitrogenase activity in a medium containing 15 mM NH_4_ (Figure 4, black bar) and activity could still be detected after 8 weeks of incubation (data not shown). Under the same growth conditions (15 mM NH_4_), the *nifV* forward mutant was unable to reduce nitrogen but this phenotype was not restored to wild-type levels by chemical or genetic complementation. When the strains were incubated under nitrogen-limiting growth conditions (0.3 mM NH_4_), both *K. sp.* M5aI and *P. phymatum* exhibited nitrogenase activity. However, the activity measured in *P. phymatum* grown with 0.3 mM NH_4_ was lower (approximately at 25%) compared to the activity of cells grown in a nitrogen-replete media (15 mM NH_4_). In nitrogen-limiting growth conditions, the *nifV* mutant strain was unable to reduce nitrogen, and nitrogenase activity could be complemented to wild-type levels when *nifV* was provided in *trans* (Figure 4, rightmost data point). However, the addition of 1 mM homocitrate to the *nifV* mutant did not restore nitrogenase activity. After the ARA test, the strains were re-isolated, and the identity and the number of colony-forming units (CFU) was determined for all strains grown with 15 and 0.3 mM NH_4_. As shown in Supplementary Table S5, a similar number of living cells (10^8^–10^10^) was recovered for all strains in both nitrogen-replete and nitrogen-limiting conditions. The results thus showed that the *P. phymatum* STM815 nitrogenase is active in free-living conditions under nitrogen-limiting and replete conditions and that this activity is dependent on the presence of *nifV*.

### 3.4. P. phymatum nifV Is Expressed in Free-Living Microoxic Conditions and in Symbiosis with Papilionoids and Mimosoids

**Figure 5:**
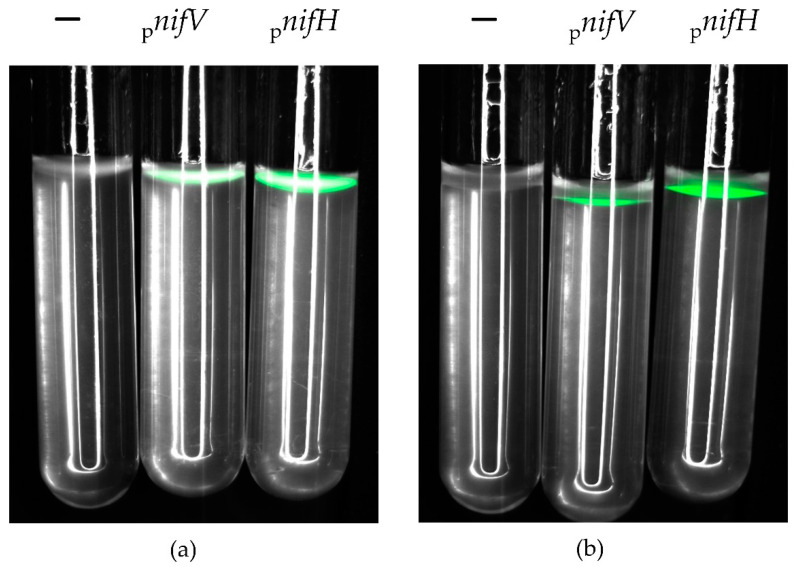
GFP expression of *P. phymatum* STM815 *nifV* and *nifH* reporter strains grown in free-living condition under 15 mM (**a**) and 0.3 mM NH_4_ (**b**) for 21 days at 28 °C. (A)BS soft agarose tubes were inoculated with different *P. phymatum* strains: wild-type carrying empty pPROBE (−), *nifV* GFP promoter fusion (_p_*nifV*) and *nifH* GFP promoter fusion (_p_*nifH*). Three independent biological replicates were inoculated per strain and condition. Images were taken 21 days after inoculation under GFP filter with 400 ms exposure. A typical result of one of the replicates is shown.

To observe *nifV* expression during free-living and symbiotic growth, a *P. phymatum* STM815 *nifV* reporter strain was constructed where the *nifV* promotor was placed in front of the gene coding for green fluorescent protein (GFP). For the expression in free-living growth conditions (Figure 5), the same settings used for the nitrogenase activity in vitro test (minimal media with succinic acid as carbon source, microoxic conditions) were used. A *nifH-gfp* reporter strain (Supplementary Table S1) was employed as a positive control and the *P. phymatum* wild-type strain harboring the empty pPROBE plasmid was used as a negative control (Supplementary Table S1). While the negative control did not show any fluorescence after 21 days, the *nifV* and *nifH* reporter strains showed expression when grown under nitrogen-replete (15 mM NH_4_Cl) and deplete (0.3 mM NH_4_Cl) conditions. Visual inspection suggested that *nifH* and *nifV* expression were higher when cells were grown under nitrogen-replete conditions (Figure 5), although the number of cells was very similar in both conditions (data not shown).

**Figure 6:**
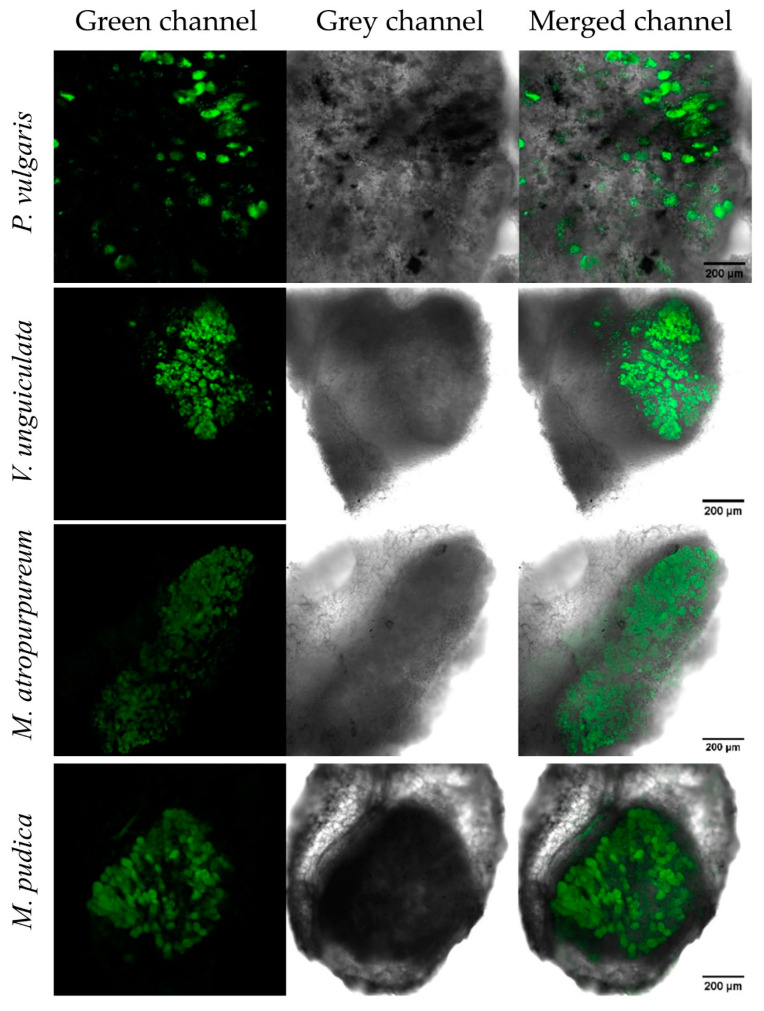
GFP expression of a *P. phymatum nifV*–GFP reporter strain in nodules of different legumes. *P. vulgaris* (common bean), *V. unguiculata* (cowpea), *M. atropurpureum* (siratro) and *M. pudica* (mimosa) nodules were analyzed 21 or 28 days post-inoculation. After the incubation, nodules were collected from the roots, sectioned, and examined under the confocal laser scanning microscope in which the GFP expression in nodules was monitored with an excitation of 488 nm. Three biological replicates of each strain were performed per host plant and one representative picture from one replicate of each type of nodule is shown.

To examine *nifV* expression during symbiosis, nodules infected by *P. phymatum* STM815 *nifV* reporter strain and a *P. phymatum* strain containing the empty pPROBE were harvested and dissected 21 dpi for common bean and cowpea and 28 dpi for siratro and mimosa. The expression of *nifV* was detected using confocal microscopy under the GFP filter. While *P. phymatum* carrying the empty pPROBE did not show any fluorescence during symbiosis with the four legumes (data not shown), *nifV* was expressed in the nodules of all four legumes (Figure 6), suggesting that *nifV* gene is important in symbiosis with the papilionoids and mimosoid plants tested.

### 3.5. Metabolic Comparison of P. phymatum STM815 Wild Type in Nodules of Different Host Plants

Host-dependent differences in nitrogenase activity of the different *nifV* mutants suggested potential metabolic differences between mimosoid and papilionoid legumes. In order to elucidate these plant-specific differences, metabolites from uninfected roots and from nodules of common bean and cowpea (21 dpi) and of siratro and mimosa (28 dpi) were extracted and analyzed using a non-targeted metabolomics approach by flow injection-time-of-flight mass spectrometry [52]. A total of 384 metabolites were identified from the root and nodule extracts. The core nodule metabolome shared by common bean, cowpea, siratro and mimosa nodules infected with *P. phymatum* STM815 wild type was identified by comparing the levels of common metabolites found in all nodules with the metabolite levels of all root samples. Of all detected metabolites, the amount of 11 metabolites was found to differ significantly between the nodule and root samples (Table 1). Specifically, eight metabolites accumulated in all nodules and three metabolites were more abundant in roots. Three metabolites involved in lysine biosynthesis and degradation (diaminopimelate, pipecolate and aminoadipate) were more abundant in nodules compared to roots. Homocitrate, which is the cofactor of the nitrogenase (see above) and also an intermediate in lysine biosynthesis, accumulated in all nodule compared to root samples (Table 1). In addition, the ion counts detected for homocitrate showed similarly high numbers (1.2–2 × 10^7^ ion intensity) in all four legumes (Supplementary Figure S4), suggesting similar amounts of this metabolite in all nodules. Moreover, we also found two intermediates of the arginine and proline metabolism (4-aminobutyraldehyde and acetyl-glutamate-semialdehyde) as significantly accumulated in nodules of all four plants. Alanine was the only common amino acid detected with increased amounts in all nodules. The metabolite 4,5-seco-Dopa, which is involved in the biosynthesis of the plant antioxidant pigment betalain [53], was also found as more abundant in all nodules. Among the three metabolites more abundant in uninoculated roots compared to nodules, we found an intermediate of the histidine metabolism (urocanate), a component of glycerophospholipids (sn-glycerol 3-phosphate) and one of the major cell wall-bound phenolic acids in plants (4-hydroxybenzoate).

A metabolite enrichment analysis also suggested that lysine biosynthesis and degradation and arginine and proline metabolism are nodule-specific metabolic pathways (Supplementary Table S6).

In order to find metabolites that show significant accumulation in one specific plant host, we compared the metabolite levels from nodules of each plant host (Supplementary Table S7). Twenty-one metabolites were more abundant in bean nodules, including isopropylmaleate, 3-propylmalate and 4-methyl-2-oxopentanoate, that are all involved in the biosynthesis of valine, leucine and isoleucine. The amino acid tyrosine was also more abundant in bean nodules. Additionally, the amount of the auxin indole-3-acetate was higher in bean nodules. Twenty-seven metabolites significantly accumulated in cowpea nodules, including acetylornithine, arginine, aspartate, asparagine, glutamate and aminobutanoic acid. The intermediates in pyrimidine biosynthesis, dihydrouracil and uracil, were also found in increased amount in cowpea nodules. Among the 12 metabolites showing increased levels in siratro nodules, we found the C4 dicarboxylates fumarate, malate and tartaric acid. Other metabolites, which accumulated in siratro nodules, were an hexuronate, hexonic acid, glucono-lactone and hydroxyadipate. Among the 12 compounds that accumulated in mimosa, we identified several metabolites involved in flavonoids and isoflavonoids biosynthesis, such as kaempferol 3-*O*-glucoside, rutin, homoeriodictyol chalcone, pratensein and vestitone.

## 4. Discussion

*Paraburkholderia phymatum* STM815 is able to grow under free-living conditions and in symbiosis with leguminous host plants. It is quite unique among the beta-rhizobial *Paraburkholderia* in that it exhibits a broad host-range able to infect both mimosoid and papilionoid legumes. In our laboratory, it has been used as a model for studying and understanding the molecular mechanisms underlying beta-rhizobial symbiosis. Our previous transcriptomics study revealed that the gene *nifV*, which is usually absent in the genomes of alpha-rhizobia, was one of the *P. phymatum* genes highly upregulated during symbiosis with *Phaseolus vulgaris* [35]. Bioinformatic and phylogenetic analyses revealed that *nifV* is present in around 50% of the genomes of the analyzed diazotrophic *Paraburkholderia* species (Supplementary Table S2) and probably originated from a common free-living diazotroph, as has been previously suggested for other *nif* genes [12,14]. All analyzed papilionoid-nodulating *Paraburkholderia* genomes contain at least two NifV copies, which seem to have a different ancestor (NifV1 has the same ancestor as the NifV found in *Paraburkholderia* nodulating mimosoids, while NifV2 resembles NifV of free-living *Paraburkholderia*) and a different position in the genome (relative to *nifA*, *nifV*1 is located upstream and *nifV2* downstream) (Figure 2). This suggests that two transfer events may have occurred. Whether the number of copies is proportional to the level of nitrogenase activity is unknown. Our results are not only intriguing from a phylogenetic point of view but also from a regulatory one, as the expression of both *nifV* copies could be controlled by different regulators. In the nitrogen-fixing cyanobacteria *Nostoc* sp. PCC7120, the expression profile of the two *nifV* copies differ spatially and temporally according to the nitrogen nutritional status of the cell [54].

We were able to determine suitable in vitro growth conditions to observe *P. phymatum* nitrogenase activity. For this, we used succinate as a carbon source and inoculated the strain in the agarose such that it could grow under microoxic conditions. In fact, a low-oxygen environment is essential for the functioning of the nitrogenase in rhizobia. Under these conditions, the *P. phymatum nifV* mutant did not show nitrogenase activity when grown in the presence of either 15 or 0.3 mM of NH_4_Cl, suggesting that homocitrate is essential for activity (Figure 4). This defect was rescued by genetic complementation under limited nitrogen growth conditions. Addition of homocitrate to the medium did not restore nitrogenase activity, possibly because of the lack of a homocitrate uptake system in *P. phymatum*. Indeed, to date, a rhizobial homocitrate transporter has not yet been identified. The higher nitrogenase activity of *P. phymatum* grown under nitrogen-replete growth conditions (Figure 4) was not due to a difference of the number of living cells in nitrogen-replete and limited conditions (Supplementary Table S5). In line with this result, previous ex planta studies have shown that *P. phymatum* needs a basal amount of nitrogen in order to produce an active nitrogenase in free-living conditions [22]. While *nifV* was transcribed under both nitrogen-replete and limited conditions at similar levels (Figure 5), we cannot exclude post-transcriptional regulatory mechanisms that may affect nitrogenase levels and/or activities under nitrogen limitation [55]. Our results in *P. phymatum* differ from what is known in other well-studied free-living diazotrophs that cannot form a symbiosis, such as *Klebsiella pneumonia* that activates the nitrogenase only under nitrogen limitation. In *K. pneumoniae*, the transcriptional regulator NtrC has been shown to activate the expression of the transcriptional regulatory gene *nifA* and its inhibitor *nifL* under nitrogen limitation. NifL inhibits NifA by direct protein–protein interaction, but under anaerobic nitrogen-limiting conditions, the PII-like protein GlnK (also controlled by NtrC) antagonizes the inhibitory effects of NifL, resulting in the activation of NifA, which then induces expression of the *nif* genes [28,51]. In *P. phymatum*, NtrC is expressed under nitrogen limitation [35] but no NifL homolog could be identified in the genome of the organism, suggesting that the regulatory networks activating *nif* gene expression under nitrogen limitation may be different from *K. pneumoniae.* Additional studies are required to elucidate the precise regulatory mechanisms underlying activation of *nifV* expression in *P. phymatum*.

In contrast to the symbioses with papilionoid host plants, where *nifV* is essential for nitrogenase functioning (Figure 3a–c), *nifV* was dispensable in *M. pudica* (Figure 3d). In fact, only mimosa nodules colonized by the *nifV* reverse mutant (where expression of the downstream genes is not supported by the promoter of the antibiotic resistance cassette, Supplementary Figure S1) did not show any nitrogenase activity. This suggests that the homocitrate level in this strain is insufficient for nitrogenase functioning and thus depends on supply by the plant (Figure 3). This idea is supported by the finding that the plant homocitrate synthase (FEN1) has been shown to be essential for symbiosis involving alpha-rhizobial strains lacking *nifV* [33,34,56]. However, in the *Aeschynomene*–photosynthetic *Bradyrhizobium* symbiosis, the lack of *nifV* was complemented only by selected plant hosts, suggesting a host plant-dependent requirement of the rhizobial *nifV* [34,56]. During the symbiosis between *Bradyrhizobium* DOA9 and *Aeschynomene americana*, homocitrate was hypothesized to also be produced by the bacterial enzyme 2-isopropylmalate synthase (LeuA), which exhibits a high amino acid identity (34%) to NifV [56]. The construction of a *P. phymatum leuA* mutant as well as a *nifV/leuA* double mutant and their phenotypic characterization in planta using different mimosoid legumes could help us to understand if LeuA is also involved in homocitrate synthesis in *P. phymatum*. A BLASTP search identified potential FEN1 candidates in the genomes of *P. vulgaris* and *V. unguiculata*, while for *M. pudica* and *M. atropurpureum*, the complete genome sequence is not available. In line with this hypothesis, homocitrate should be transported across the symbiosome and bacteroid membrane. However, to this date, it is still unclear how the homocitrate produced by the plant is transported from the plant to the bacteroid and how this transport mechanism is regulated. The residual nitrogenase activity of papilionoids nodules infected with both *nifV* mutants suggested the presence of an alternative ligand for FeMo-co in the catalytic center of the nitrogenase. Previous crystallographic analysis of *K. pneumoniae* nitrogenase showed that citrate, which differs from homocitrate by a single methylene group, is also able to bind to FeMo-co [57]. The use of an alternative ligand and the expression of the *nifV* downstream genes (*nifW* and *fixABC*X) may also explain why nodules infected by the *nifV* forward mutant still showed residual nitrogenase activity (2% to 6% that of wild-type nodules).

To identify both the common and specific metabolic pathways occurring in nodules formed by *P. phymatum* in the different papilionoid and mimosoid plants, we compared the relative metabolite abundance from uninoculated roots and *P. phymatum*-induced nodules. To the best of our knowledge, this is the first study where the global metabolite profile of a beta-rhizobial strain was measured in different host plants. Notably, we found that homocitrate was one out of eight metabolites detected in all nodules at increased levels compared to uninfected roots, confirming its importance during *P. phymatum*-legumes symbioses. However, we were not able to distinguish the origin (plant or rhizobia) of most of the detected metabolites [58]. Further metabolomic experiments using nodules induced by a *nifV* mutant would help us to understand the bacterial contribution to the accumulation of homocitrate in the nodules. Homocitrate is also involved in the biosynthesis of lysine [59] and several metabolites linked to lysine biosynthesis and degradation (diaminopimelate, pipecolate and aminoadipate) were also shown to occur at higher levels in nodules compared to uninfected roots. This result suggests that lysine plays an important role during beta-rhizobial symbiosis. In line with these results, previous studies showed that *lysA* mutants of *Rhizobium etli* and *Mesorhizobium cicero* are unable to form functional nodules in several legumes [60,61]. Finally, the concentration of metabolites involved in arginine and proline biosynthesis (4-aminobutyraldehyde and acetyl-glutamate-semialdehyde) as well as the amino acid alanine were found to be increased in nodules. Arginine, which can be metabolized to proline, has been shown to be involved in the early infection step and is required as a carbon and nitrogen source in alpha-rhizobia [62]. Interestingly, a new model of nitrogen exchange called CATCH-N cycle (for C4-dicarboxylate arginine transamination co-catabolism under acidic (H+) conditions to fix nitrogen) has been recently described for *Sinorhizobium meliloti* and *Bradyrhizobium diazoefficiens* symbiosis [63]. According to this model, bacteroids take up succinate and arginine and secrete aspartate and alanine in a balanced two-way exchange of C4-dicarboxylates, protons and amino acids. After translocation into the legume cytosol, alanine and/or ammonia and aspartate are assimilated into ureides or amides depending if the nodules are determinate or indeterminate, respectively [64]. Among the metabolites that were accumulating in all uninfected roots, we found the cell wall-bound phenolic acid compound 4-hydroxybenzoate, which is known to play a major role in plant defense against pathogens [65]. A closer look into host-specific metabolites showed that mimosa nodules infected with *P. phymatum* accumulated several metabolites related with flavonoids and isoflavonoids biosynthesis. Different roles have been attributed to flavonoids/isoflavonoids, including the induction of Nod factors’ production [66], protection against pathogens [67], antioxidant properties [68] and additionally, they can be used as a carbon source by rhizosphere microorganisms [69]. In line with our results suggesting that the host plant mimosa may assist in the supply of homocitrate for optimal nitrogenase activity, this plant may secrete more flavonoids in order to attract more rhizobia and hence obtain more fixed nitrogen [70].

In summary, this study revealed an important role of the rhizobial homocitrate synthase (NifV) in the symbiosis between *P. phymatum* and agriculturally important papilionoid legumes. The presence of several NifV copies in the genome of papilionoid-nodulating strains may indicate that these strains display higher nitrogenase activity in nodules. Whether high homocitrate levels in the nodules could be engineered to increase the symbiotic efficiency remains to be investigated. However, such an approach could be of enormous ecological and agronomical importance for future crop production without the use of chemical fertilizers.

## Figures and Tables

**Table 1:** List of 11 metabolites that significantly accumulated in *P. vulgaris* (bean), *V. unguiculata* (cowpea), *M. atropurpureum* (siratro) and *M. pudica* nodules induced by *P. phymatum* STM815 wild-type (wt nod) compared to the non-root samples (root).

Metabolites ^1^	ID ^1^	log_2_FC (Bean wt nod vs. Root) ^2^	log_2_FC (Cowpea wt nod vs. Root) ^2^	log_2_FC (Siratro wt nod vs. Root) ^2^	log_2_FC (Mimosa wt nod vs. Root) ^2^
**Higher Abundance in Nodules Infected by *P. phymatum* Wild-Type**					
Diaminopimelate	C00666	0.7	1.1	1.9	1.2
Aminoadipate	C00956	0.8	0.8	1.7	1.3
4-aminobutyraldehyde	C00555	0.7	0.9	1.6	1.0
Alanine	C00041	0.6	1.1	1.6	1.0
Homocitrate	C01251	0.6	1.3	1.4	1.2
4,5-seco-Dopa	C17758	0.5	0.7	1.3	1.0
Acetyl-Glu-semialdehyde	C01250	1.2	1.1	1.0	1.6
Pipecolate	C00408	0.8	0.9	0.8	0.9
**Higher Abundance in Uninfected Roots**					
Urocanate	C00785	−0.7	−0.7	−1.4	−0.5
sn-glycerol 3-phosphate	C00093	−1.5	−0.6	−1.5	−0.9
4-hydroxybenzoate	C00156	−0.5	−0.6	−0.7	−0.8

^1^ Metabolite name and ID according to the Kyoto Encyclopedia of Genes and Genomes (KEGG) database; ^2^ log_2_ of the metabolite level fold change (FC), comparing nodules induced by wild-type (wt nod) with non-inoculated roots (root) for each single plant (bean, cowpea, siratro and mimosa). Dopa: dihydroxy-phenylalanine, Glu: Glutamate.

## Data Availability

Not applicable.

## Supplementary Materials

The following Supplementary Materials are available online at https://www.mdpi.com/article/10.3390/cells10040952/s1, Figure S1: Illustration of the direction of the chloramphenicol resistance cassette (Cm^R^) in the *nifV* forward and *nifV* reverse mutant strains. The genes (Bphy_7741-35 operon) are represented as arrows; Figure S2: Maximum likelihood phylogenetic tree of nitrogen-fixing beta-proteobacteria strains based in 16S rRNA nucleotide sequences. Colored boxes indicate the different *Paraburkholderia* groups. Bootstrap test values are expressed as percentages and values are shown if greater than 50%. The asterisks indicate the exception inside the nodulating *Paraburkholderia* group (*) and the cross the exception inside the free-living *Paraburkholderia* group (†); Figure S3: Colony-forming units (CFU) of *P. phymatum* wild-type, *nifV* forward and *nifV* reverse mutant bacteroids isolated from nodules of *P. vulgaris, V. unguiculata, M. atropurpureum* and *M. pudica*. The bacteroids were isolated from at least one biological replicate and were plated in LB without salt and in selective LB without salt for counting; Figure S4: Comparison of the average ion intensities detected for homocitrate in *P. vulgaris* (bean), *V. unguiculata* (cowpea), *M. atropurpureum* (siratro) and *M. pudica* (mimosa) non-inoculated roots (not) and in nodules induced by *P phymatum* STM815 (wt). Three biological replicates were examined (with the exception for *P. vulgaris*, where only two replicates were used), each analyzed twice by non-target metabolomics. Table S1: Bacterial strains, plasmids and oligonucleotides used in this work; Table S2: References and accession numbers for all the strains used in the phylogenetic analysis; Table S3: BLASTP results in the *Paraburkholderia* group presenting at least a 70% of identity and an e-value lower than 1 × 10^−5^ to *P. phymatum* NifV protein sequence. The comparison with the annotated NifV amino acid sequences from the nitrogen-fixing strains *Burkholderia vietnamiensis* LMG 10929, *Trinickia symbiotica* JPY-345, *Klebsiella* sp. M5aI and *Bradyrhizobium* sp. ORS285 are also shown in this analysis; Table S4: Raw metabolomics data including injections, ions annotations and intensities. Table S5: Number of colony-forming cells (CFU) obtained from strains re-isolated after 64 days of inoculation in nitrogen-replete (15 mM NH_4_Cl) and nitrogen-limiting (0.3 mM NH_4_Cl) growth media. Table S6: Enrichment analysis of KEGG pathways in metabolites detected in *Phaseolus vulgaris* (bean), *Vigna unguiculata* (cowpea), *Macroptilium atropurpureum* (siratro) and *Mimosa pudica* (mimosa) nodules induced by *P. phymatum* STM815 wild-type (wt) compared to uninoculated roots (roots). The enrichment analysis was performed using the bacteria and plant KEGG database; Table S7: Host-specific metabolites showing increased levels in nodules of one out of the four tested plants. The log_2_ fold change of the metabolite level in *Phaseolus vulgaris* (bean) or *Vigna unguiculata* (cowpea) or *Macroptilium atropurpureum* (siratro) or *Mimosa pudica* (mimosa) nodules induced by *P. phymatum* STM815 wild-type (wt nod) compared to uninoculated roots (root) is given.
