# Supplementary material for: Paraburkholderia phymatum Homocitrate Synthase NifV Plays a Key Role for Nitrogenase Activity during Symbiosis with Papilionoids and in Free-Living Growth Conditions
Source: Cells. 2021 Apr 20;10(4):952. doi: 10.3390/cells10040952 (PMC8073898; doi:10.3390/cells10040952)
Supplement: Supplementary file 1 [file cells-10-00952-s001.zip › Belles et al_Table S1.pdf]

Table S1: Bacterial strains, plasmids and oligonucleotides used in this work

Strain or plasmid	Description	Reference
Strains		
Escherichia coli		
cc118λ-pir	Δ(ara-leu) *araD* Δ*lacX74 galE galK phoA20 thi1 rpsE rpoB argE(Am) recAl* λ pir; $Strep^R$	[1]
Top10	Δ*lacX74 ara* Δ*139*Δ(*ara-leu*)	Invitrogen
Paraburkholderia phymatum		
STM815	Wild type	[2]
Δ*nifV*_F	Δ*nifV* of STM815; Cm^R	This study
Δ*nifV*_R	Δ*nifV* of STM815; Cm^R	This study
Δ*nifV*_F-comp	STM815-Δ*nifV*_F mutant harboring pBBR1MCS-5-*nifV*; Km^R	This study
WT-pPROBE	STM815 harboring pPROBE-NT empty vector; Km^R	[3]
WT-pPROBE-*nifV*	STM815 harboring pPROBE-NT with the promoter of *nifV*	This study
WT-pPROBE-*nifH*	STM815 harboring pPROBE-NT with the promoter of *nifH*	This study
Klebsiella pneumoniae 5022	*K.* sp. M5aI strain, *his*D2 genotype	[4]
Plasmids		
pBBR1MCS-2	Broad host-range cloning vector; Km^R	[5]
pEX18Tc	Suicide plasmid; Tc^R	[6]
pPROBE-NT	Broad-host-range promoter-probe vector; Km^R	[7]
pRK2013	Helper plasmid; Km^R	[8]
pSHAFT2	Suicide plasmid; Cm^R	[9]
pBBR1MCS-2-*nifV*	pBBR1MCS2 containing *nifV* (Bphy_7741) for complementation; Km^R	This study
pEX18Tc- *nifV*_F	pEX18Tc-containing a 485 bp upstream fragment and a 479 bp downstream fragment of *nifV* for mutagenesis; Tc cassette in forward direction, Tc^R	This study
pEX18Tc- *nifV*_R	pEX18Tc-containing a 485 bp upstream fragment and a 479 bp downstream fragment of *nifV* for mutagenesis; Tc cassette in reverse direction, Tc^R	This study
pPROBE- *nifV*	pPROBE-NT containing the *nifV* promoter (431 bp); GFP expressing promoter reporter, Km^R	This study
**Oligonucleotides**	**Sequence[1]**	**Source**
Bphy7741_up_F_EcoRI	GCGCgaattcGATGCCGATACAGGCAGTTG	This study
Bphy7741_up_R_NdeI	CGCGcatatgCGGTTTGAGCATGTCTACGG	This study
Bphy7741_down_F_NdeI	CGCGcatatgGAGGCGTCATGAGTGTGCT	This study
Bphy7741_down_R_EcoRI	GCGCgaattcGGTGTTGTTGACTGGGTGAA	This study
Bphy7741_veri	TCCAGCGAGAACAGATCGTA	This study
Bphy7742_veri	TGCGATTGCAGTAGTTGCAC	This study
Bphy7741_comp_F_XbaI	GCGCtctagaGATCAGGTTTCTCGGGTTTG	This study
Bphy7741_comp_R_HindIII	GCGCaagcttTCATGACGCCTCCACTCG	This study
Bphy7741_prom_EcoRI_F	GCGCgaattccGTCTACGGCCTATTGAATAGT	This study
Bphy7741_prom_SalI_R	GCGCgtcgacGGATATCTCCTCGCAACCGAC	This study

catA2_F_NdeI	GCGCcatatgTTGACAATTAAGCCCGTATATGG	This study
catA2_R_NdeI	GCGCcatatgCCGGATACGGTGGCTTAAAT	This study
Bphy7808_nifH_F	GGCGTGGACTATGTGTCGTA	[10]
Bphy7808_nifH_R	GATGCCCTTCGAGATGTTGT	[10]
Koxy_nifV_F	ATCCATCTTGCGCATACCCT	This study
Koxy_nifV_R	GTGGTAAAAGGGTCGAGCAG	This study

[1]restriction sites are in lower letters.

References

1. Herrero, M.; De Lorenzo, V.; Timmis, K.N. Transposon vectors containing non-antibiotic resistance selection markers for cloning and stable chromosomal insertion of foreign genes in gram-negative bacteria. *J. Bacteriol.* **1990**, *172*, 6557–6567, doi:10.1128/jb.172.11.6557-6567.1990.

2. Moulin, L.; Munive, A.; Dreyfus, B.; Boivin-Masson, C. Nodulation of legumes by members of the beta-subclass of Proteobacteria. *Nature* **2001**, *411*, 948, doi:https://doi.org/10.1038/35082070.

3. Liu, Y.; Bellich, B.; Hug, S.; Eberl, L.; Cescutti, P.; Pessi, G. The exopolysaccharide cepacian plays a role in the establishment of the *Paraburkholderia phymatum – Phaseolus vulgaris* symbiosis. *Front. Microbiol.* **2020**, *11*, 1–13, doi:10.3389/fmicb.2020.01600.

4. Cali, B.M.; Micca, J.L.; Stewart, V. Genetic regulation of nitrate assimilation in *Klebsiella pneumoniae* M5al. *J. Bacteriol.* **1989**, *171*, 2666–2672, doi:10.1128/jb.171.5.2666-2672.1989.

5. Elzer, P.H.; Roop, R.M.; Kovach, M.E.; Robertson, G.T.; Peterson, K.M.; Steven Hill, D.; Farris, M.A. Four new derivatives of the broad-host-range cloning vector pBBR1MCS, carrying different antibiotic-resistance cassettes. *Gene* **1995**, *166*, 175–176, doi:10.1016/0378-1119(95)00584-1.

6. Hoang, T.T.; KarkhoV-Schweizer, R.R.; Kutchma, A.J.; Schweizer, H.P. A broad-host-range Flp-*FRT* recombination system for site-specificexcision of chromosomally-located DNA sequences: application forisolation of unmarked *Pseudomonas aeruginosa* mutants. *Gene* **1998**, *212*, 77–86.

7. Miller, W.G.; Leveau, J.H.J.; Lindow, S.E. Improved *gfp* and *inaZ* broad-host-range promoter-probe vectors. *Mol. Plant-Microbe Interact.* **2000**, *13*, 1243–1250, doi:10.1094/MPMI.2000.13.11.1243.

8. Phadnis, S.H.; Berg, D.E. Identification of base pairs in the outside end of insertion sequence IS*50* that are needed for IS*50* and Tn*5* transposition. *Proc. Natl. Acad. Sci. U. S. A.* **1987**, *84*, 9118–9122, doi:10.1073/pnas.84.24.9118.

9. Shastri, S.; Spiewak, H.L.; Sofoluwe, A.; Eidsvaag, V.A.; Asghar, A.H.; Pereira, T.; Bull, E.H.; Butt, A.T.; Thomas, M.S. An efficient system for the generation of marked genetic mutants in members of the genus *Burkholderia*. *Plasmid* **2017**, *89*, 49–56, doi:10.1016/j.plasmid.2016.11.002.

10. Lardi, M.; Liu, Y.; Purtschert, G.; de Campos, S.B.; Pessi, G. Transcriptome analysis of *Paraburkholderia phymatum* under nitrogen starvation and during symbiosis with *Phaseolus vulgaris*. *Genes (Basel).* **2017**, *8*, doi:10.3390/genes8120389.
