# Supplementary figures and images for: Paraburkholderia phymatum Homocitrate Synthase NifV Plays a Key Role for Nitrogenase Activity during Symbiosis with Papilionoids and in Free-Living Growth Conditions
Source: Cells. 2021 Apr 20;10(4):952. doi: 10.3390/cells10040952 (PMC8073898; doi:10.3390/cells10040952)

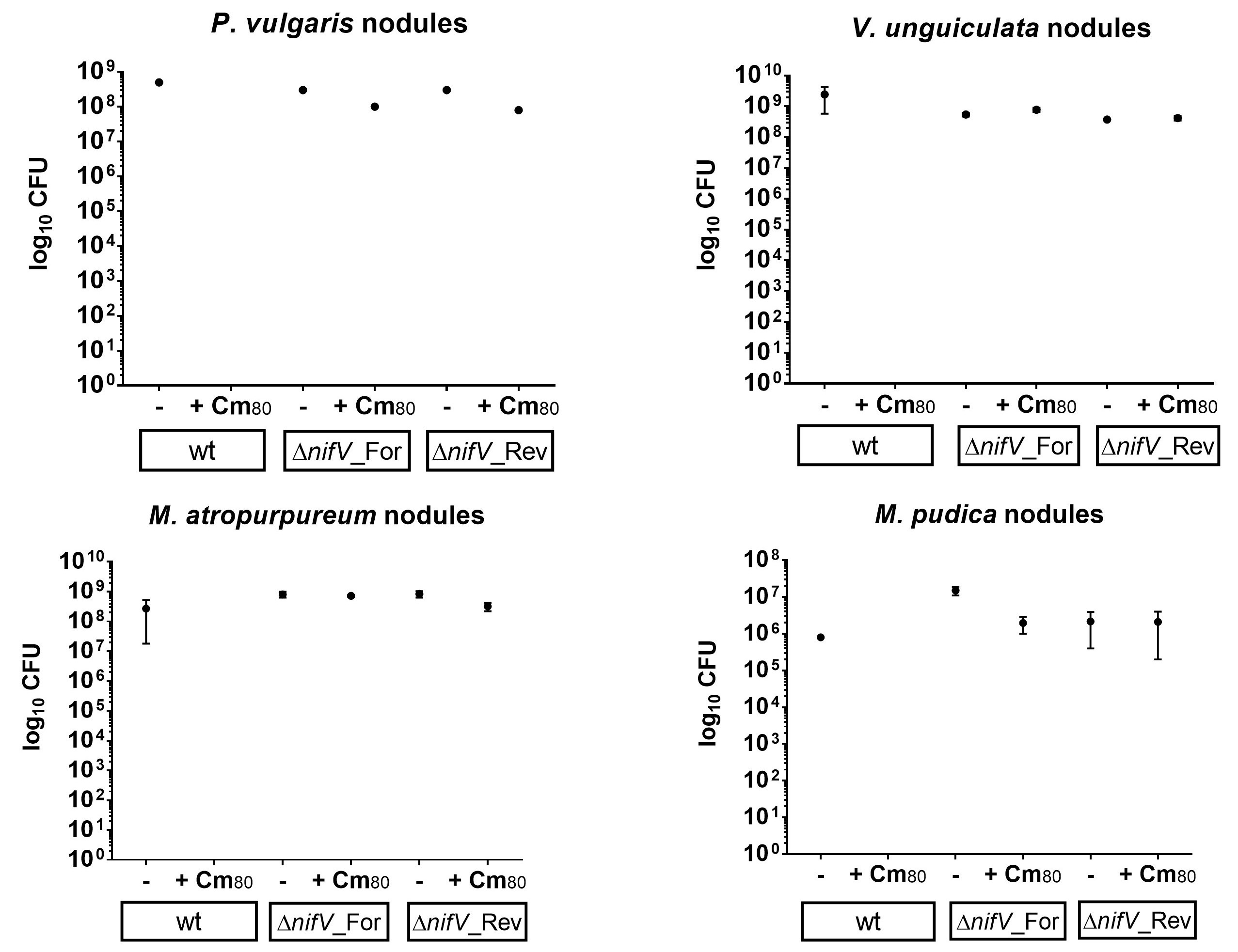

Supplement: Supplementary file 1 [file cells-10-00952-s001.zip › Figure S3_revised 20210420.jpg]

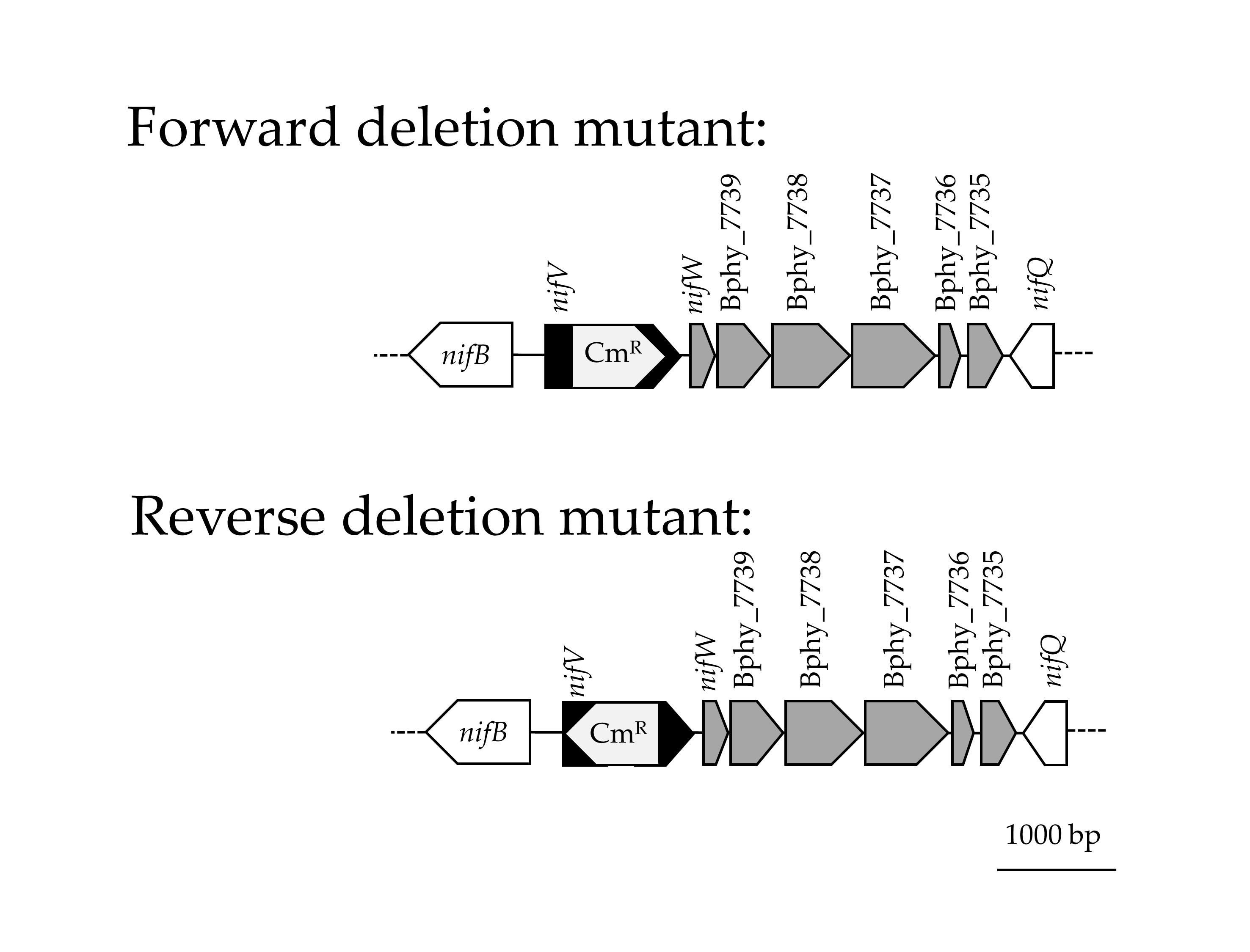

Supplement: Supplementary file 1 [file cells-10-00952-s001.zip › Figure_S1_300dpi.jpg]

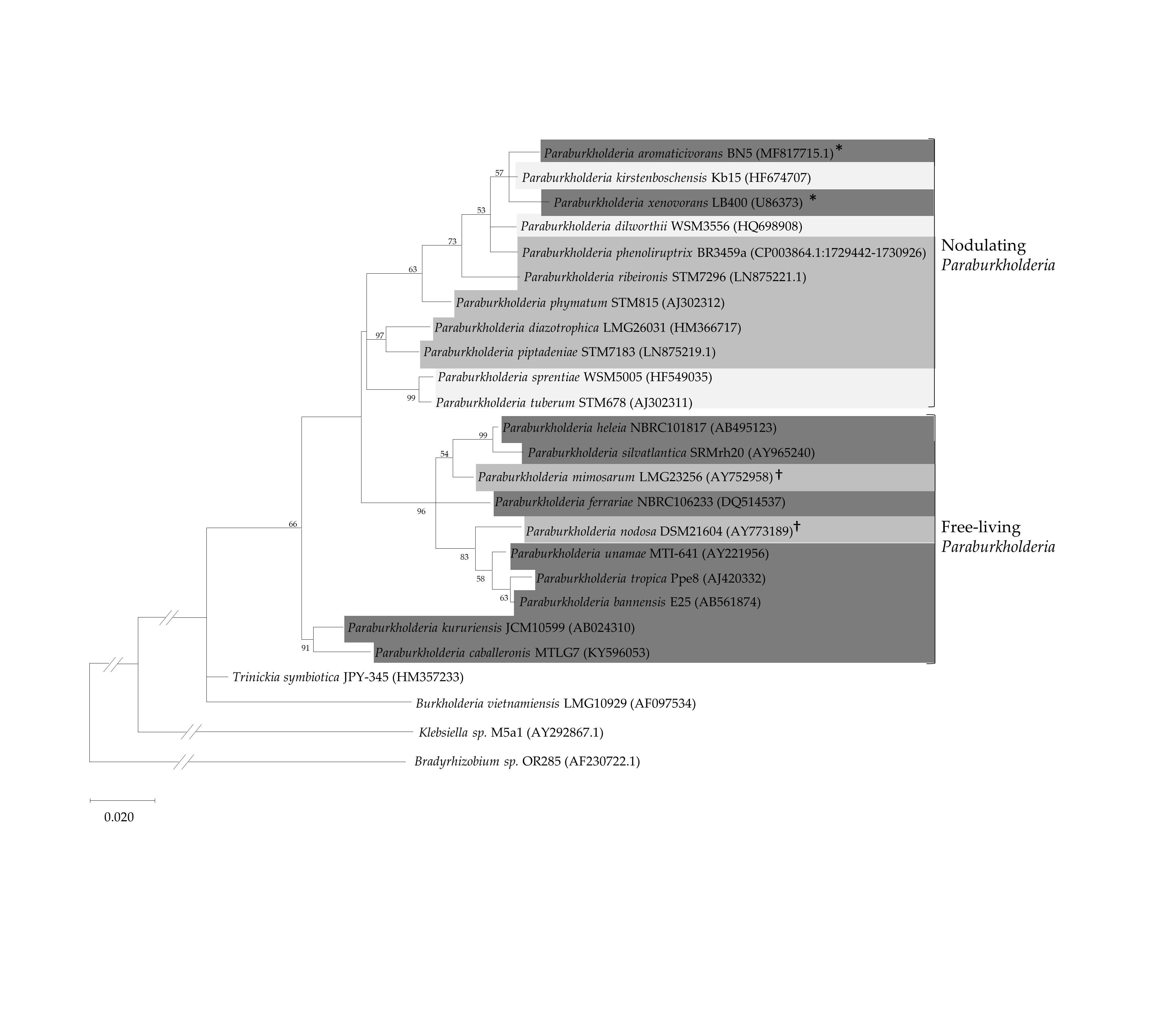

Supplement: Supplementary file 1 [file cells-10-00952-s001.zip › Figure_S2_300dpi.jpg]

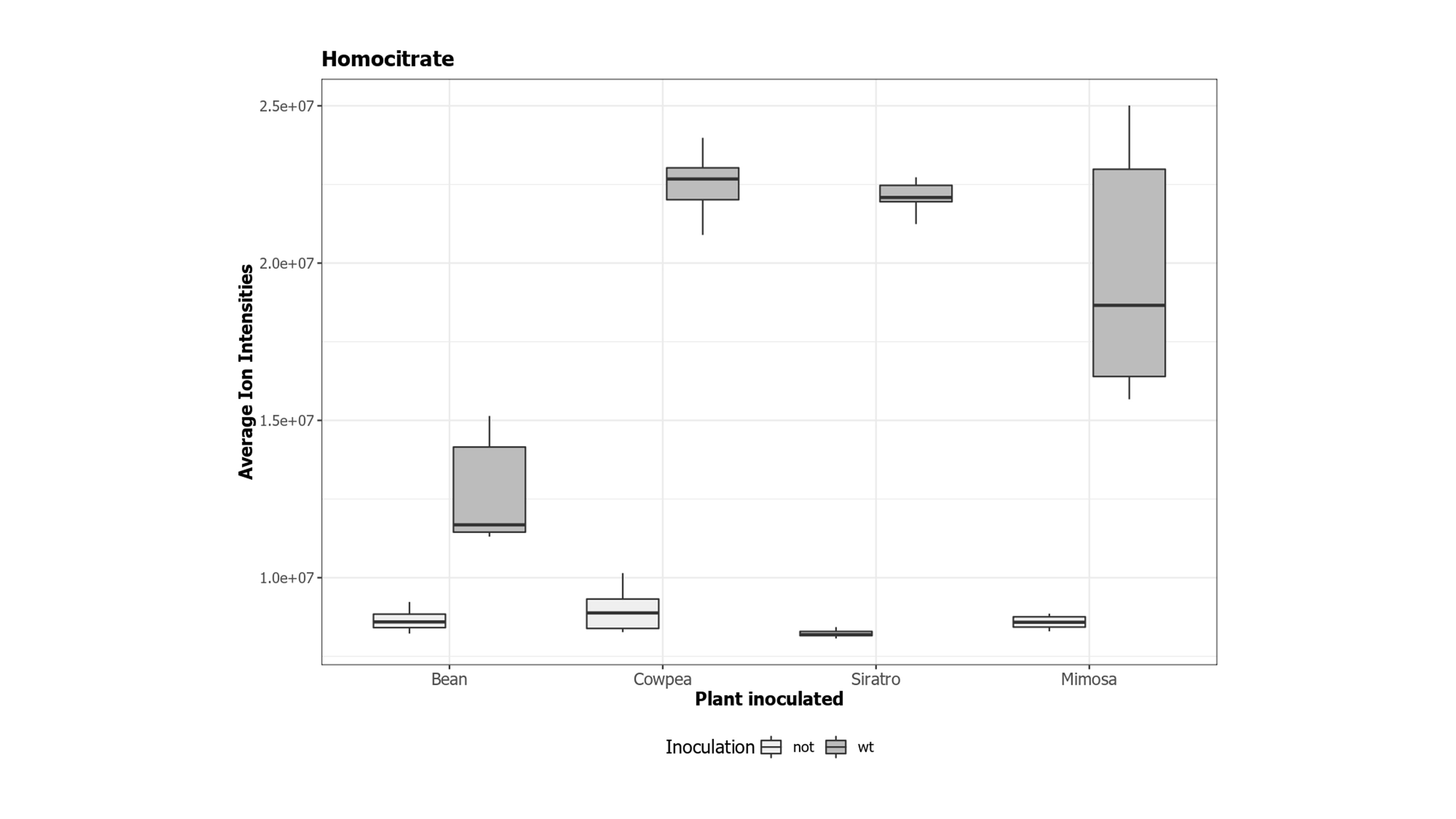

Supplement: Supplementary file 1 [file cells-10-00952-s001.zip › Figure_S4_300dpi.jpg]
